# Investigation of the most common perinatal and postnatal risk factors in children with MIH compared to healthy (non-MIH) children aged 6–12 years in Arak City

**DOI:** 10.1186/s12903-025-06948-6

**Published:** 2025-10-09

**Authors:** Hedieh Moradi, Yasmin Sheikhhassani, Zahra Sajadi, Malihe Safari

**Affiliations:** 1 Dentistry School, Arak University of Medical Sciences, Arak, Iran; 2 Department of Biostatistics, Medicine School, Arak University of Medical Sciences, Arak, Iran

**Keywords:** Molar-Incisor hypomineralization, Perinatal factors, Postnatal factors, Children, Enamel defects

## Abstract

**Background:**

Molar-Incisor Hypomineralization (MIH) is a widespread developmental enamel defect impacting children globally, with a multifactorial etiology that remains incompletely understood. Its rising prevalence presents significant challenges to oral health and quality of life. This study examines both perinatal and postnatal risk factors associated with MIH in a pediatric population.

**Methods:**

This case-control study involved 426 children aged 6–12 years, with 213 diagnosed with MIH and 213 healthy controls, recruited from a university dental clinic and local schools in Arak City, Iran. Parents completed a validated 48-item checklist covering perinatal factors (e.g., delivery mode, preterm birth) and postnatal factors (birth to 3 years, e.g., feeding practices, illnesses, medication use). MIH was diagnosed using the European Academy of Paediatric Dentistry criteria. Data were analyzed using chi-square tests and multivariate logistic regression to identify risk factors, with a significance level of 5%. Ethical approval and informed consent were obtained.

**Results:**

Vaginal delivery (42.9% vs. 62.9%, *p* = 0.004) was protective against MIH (OR = 0.54, *p* = 0.037), reducing odds by 46%, while preterm birth (18.1% vs. 8.6%, *p* = 0.048) showed a modest association. Frequent analgesic use (65.7% vs. 57.4%, *p* = 0.034) and recurrent diarrhea (37.0% vs. 32.4%, *p* = 0.033) were more prevalent in the MIH group, though effects were weak. Although a history of chickenpox was more frequent in the MIH group (31.5% vs. 17.6%, *p* = 0.058 in univariate analysis), multivariate logistic regression, adjusting for confounders, showed that children with a chickenpox history had lower MIH risk (OR = 2.12 for absence of chickenpox, *p* = 0.040). Vitamin D deficiency (6.5% vs. 8.3%, *p* = 0.829) and breastfeeding < 6 months (4.6% vs. 8.3%, *p* = 0.700) showed no clear link. Hypoxia at birth (6.7% vs. 3.8%, *p* = 0.353) and high fever (9.3% vs. 7.4%, *p* = 0.953) were not associated with MIH.

**Conclusion:**

Vaginal delivery and chickenpox history emerged as protective factors against MIH, while preterm birth, analgesic use, and diarrhea showed modest associations. These findings underscore MIH’s complex etiology, with regional variations suggesting diverse influences. Further longitudinal research is needed to validate these relationships and guide targeted prevention strategies.

## Background

Molar-Incisor Hypomineralization (MIH) is a developmental dental defect characterized by demarcated opacities, enamel breakdown, and increased caries susceptibility in the first permanent molars and incisors [1]. MIH affects approximately 10–20% of children globally, with prevalence varying by region and population [2]. The condition poses significant clinical challenges due to its association with hypersensitivity, rapid progression of dental caries, and aesthetic concerns, often necessitating extensive restorative interventions [3]. Despite its growing recognition, the etiology of MIH remains multifactorial and incompletely understood, with evidence suggesting a complex interplay of genetic, environmental, and systemic factors during the critical periods of enamel formation [4].

Enamel development, which begins in utero and continues into early childhood, is highly sensitive to disruptions [5]. Perinatal factors, such as preterm birth, mode of delivery, and maternal health conditions like preeclampsia, have been implicated as potential contributors to MIH by affecting ameloblast function during the prenatal and early postnatal phases [6]. For instance, studies have linked preterm birth and low birth weight to enamel defects, possibly due to hypoxic stress or nutritional deficiencies in the neonatal period. Similarly, postnatal factors—including early childhood illnesses, medication use, and nutritional practices—may exacerbate susceptibility to MIH by altering enamel mineralization processes [7]. Frequent use of analgesics or antibiotics, as well as infections such as chickenpox, have been hypothesized to influence MIH development, though findings remain inconsistent across studies [8].

The clinical and public health implications of MIH underscore the need to identify modifiable risk factors to inform preventive strategies [9]. Previous research has often focused on isolated factors, such as prenatal complications or postnatal infections, without integrating these dimensions into a cohesive framework [10]. This fragmented approach limits our understanding of how perinatal and postnatal events collectively contribute to MIH. Moreover, the protective or aggravating roles of specific factors, such as delivery mode or early viral exposures, remain underexplored [11].

Moreover, the protective or aggravating roles of specific factors, such as delivery mode or early viral exposures, remain underexplored [12]. Cesarean delivery, increasingly common worldwide, may alter early microbial colonization and immune development, potentially influencing enamel formation, yet its role in MIH is rarely examined systematically [13]. Similarly, viral infections like chickenpox, which peak during the critical period of molar mineralization, could either disrupt or, intriguingly, protect against enamel defects through immune-mediated mechanisms, a hypothesis that warrants further investigation [14]. Addressing these gaps is critical, particularly as MIH prevalence appears to be rising in some populations, potentially reflecting changes in obstetric practices or childhood health patterns.

This study aims to comprehensively evaluate the perinatal and postnatal influences on MIH in a cohort of 426 children aged 6–12 years, comparing those with MIH to unaffected controls. By integrating demographic characteristics with a broad spectrum of risk factors—including preterm birth, delivery mode, breastfeeding practices, medication use, and childhood illnesses—we seek to elucidate the multifactorial etiology of MIH. Our findings may provide insights into potential preventive measures and guide future research into the mechanisms underlying this prevalent dental condition.

## Methods

### Materials and methods

#### Study design and population

This case-control study was conducted in Arak City, Iran, between September 2024 and January 2025, targeting children aged 6–12 years to investigate perinatal and postnatal risk factors associated with Molar-Incisor Hypomineralization (MIH). The age range of 6–12 years was selected because it corresponds to the eruption period of first permanent molars (typically between 6 and 7 years) and incisors (6–8 years), allowing clinical assessment of MIH, while minimizing secondary damage like caries or restorations that may obscure diagnosis in older children. A total of 426 participants were recruited from two primary settings: the University Clinic of Dentistry at the Medical University of Arak (*n* = 246) and four randomly selected public schools in Arak (*n* = 180). At the schools, initial screening was conducted by trained dental hygienists under the supervision of the principal investigator, using visual inspection with disposable dental mirrors under natural lighting to identify children with potential enamel defects or dental anomalies. Children flagged during screening were referred to the university dental clinic for definitive examination by three calibrated pediatric dentists, who confirmed MIH diagnoses using standardized tools (e.g., dental mirrors, explorers, artificial lighting). The MIH group comprised 213 children diagnosed with MIH during routine dental examinations, while the control group included 213 age- and sex-matched healthy (non-MIH) children with no evidence of structural enamel defects or systemic conditions affecting dental development. Inclusion criteria for the MIH group required at least one first permanent molar exhibiting hypomineralization, with or without incisor involvement, based on the European Academy of Paediatric Dentistry (EAPD) criteria (2003, updated 2021) [15]. The EAPD criteria were chosen for their standardized, practical application in clinical and school settings, ensuring reliable diagnosis of MIH presence with high inter-examiner consistency (Cohen’s kappa = 0.89). While the Ghanim et al. (2015) criteria, endorsed by the EAPD for detailed enamel defect classification, offer specificity for epidemiological studies, they were not used due to their complexity and the study’s focus on MIH diagnosis rather than defect severity [16]. For 6-year-olds, where first permanent molars may be partially erupted, examinations focused on visible molar surfaces using standardized lighting, dental mirrors, and explorers, with children excluded if no first permanent molars were sufficiently erupted for MIH assessment, ensuring diagnostic accuracy. Exclusion criteria included congenital dental anomalies (e.g., amelogenesis imperfecta), systemic diseases known to affect enamel development, including celiac disease, hypophosphatemic rickets, chronic kidney disease, and severe metabolic disorders (e.g., phenylketonuria), or incomplete parental consent. These systemic diseases were excluded because they can cause enamel defects similar to MIH, such as hypomineralization or hypoplasia, through mechanisms like mineral malabsorption or disrupted calcium/phosphate metabolism, which could confound the association between investigated risk factors and MIH. Participants were recruited consecutively from clinical and school settings to ensure a representative sample reflective of the local population.

#### Sample size

Sample size was calculated using G*Power software (version 3.1) with an alpha of 0.05, power of 80%, and an expected odds ratio of 2.0 for key risk factors (e.g., preterm birth, analgesic use), based on prior MIH studies [17]. This yielded a minimum of 200 participants per group, adjusted to 213 per group (total *n* = 426) to account for potential dropouts and to enhance statistical power for detecting smaller effect sizes across multiple variables. Participants were matched by age (± 6 months) and sex to minimize confounding. Due to the reliance on maternal recall and cross-verification with medical or school health records, complete data for certain perinatal (e.g., delivery mode, preterm birth) and postnatal (e.g., analgesic use, chickenpox) risk factors were available for a subset of participants (105 for perinatal factors and 108 for postnatal factors per group). This reduction in assessed sample size for specific variables was due to incomplete or unverifiable records for some participants, which is addressed in the Limitations section. The subsets of 105 or 108 per group still provided sufficient statistical power for the analyses of these specific risk factors, exceeding the minimum required sample size.

#### Data collection

Data on perinatal and postnatal risk factors were collected using a comprehensive 48-item checklist, developed and validated in a pilot study involving 30 mothers (Cronbach’s α = 0.85). The checklist, completed by the children’s mothers during face-to-face interviews, covered two main domains: [1] perinatal factors, including delivery mode (vaginal vs. cesarean), gestational age (preterm ≤ 37 weeks vs. term), birth weight (≤ 2500 g vs. >2500 g), maternal complications (e.g., preeclampsia), and neonatal conditions (e.g., hypoxia, incubator use); and [2] postnatal factors from birth to three years, encompassing feeding practices (e.g., breastfeeding duration, formula use), medical history (e.g., recurrent high fever > 39 °C, infections like chickenpox or otitis media), medication use (e.g., amoxicillin, analgesics, antihistamines), and environmental exposures (e.g., hospital admissions). To enhance the reliability of maternal recall for events occurring 3–9 years prior, the checklist used structured, specific questions with clear timeframes (e.g., “during the first three years of your child’s life”), and interviews were conducted by trained interviewers to minimize misunderstanding. Cross-verification was performed with pediatric or obstetric medical records for approximately 60% of participants, where available, covering factors like delivery mode, preterm birth, and infections, while school health logs supplemented data on vaccinations and illnesses (e.g., chickenpox) for about 80% of school-recruited participants. The “birth to three years” age range was selected because it corresponds to the critical period of enamel mineralization for the first permanent molars and incisors, which are affected in MIH, with mineralization typically completing by approximately 3 years of age [18]. This timeframe captures exposures most likely to disrupt ameloblast function, as supported by prior MIH research [19], and ensures reliable maternal recall for early childhood events. Responses were recorded as categorical (yes/no/unknown), ordinal (e.g., duration in months), or continuous (e.g., birth weight in grams), with a clearly defined recall period to reduce bias. Vitamin D deficiency was assessed via maternal reports of a clinical diagnosis by a pediatrician or documented evidence in medical records, defined as a serum 25-hydroxyvitamin D [25(OH)D] level below 20 ng/mL (50 nmol/L) during the first three years of life, based on international guidelines [20]. No blood samples were collected for vitamin D measurement due to the retrospective design and logistical constraints. Clinical data were cross-verified with available medical records from pediatric or obstetric files where accessible, and maternal recall was supplemented with school health logs for consistency. Due to incomplete or unverifiable records for some participants, certain risk factors (e.g., preterm birth, vaginal delivery, analgesic use, chickenpox) were assessed in a subset of 105 (perinatal factors) or 108 (postnatal factors) participants per group, as reflected in Tables 2 and 3. This reduction in assessed sample size for specific variables did not compromise the overall study power, as the subsets remained above the minimum required sample size.

#### Data analysis

Statistical analyses were conducted using SPSS version 24 (IBM Corp., Armonk, NY). Descriptive statistics summarized participant characteristics and risk factor prevalence as means ± standard deviations (SD) for continuous variables and frequencies with percentages for categorical variables. Normality of continuous variables (e.g., age, maternal age, breastfeeding duration) was assessed using the Shapiro-Wilk test. Between-group differences in quantitative variables were analyzed using the independent t-test for normally distributed data or the Mann-Whitney U test if normality assumptions were violated. Qualitative variables (e.g., delivery mode, infection history) were compared using the chi-square test or Fisher’s exact test for expected cell counts below 5. Effect sizes were reported using Cramér’s V or Phi coefficients to quantify association strength. To identify significant risk factors for MIH, binary logistic regression was performed with MIH status (yes/no) as the dependent variable. Univariate analyses screened variables with *p* < 0.25 for inclusion in a multivariate model, adjusted for potential confounders (e.g., sex, maternal age, socioeconomic status inferred from parental education). To account for the matched design, age and sex, used as matching variables, were included as covariates in the multivariate logistic regression model to control for residual confounding, ensuring that the associations between risk factors and MIH were not biased by these factors. The significance level was set at 5% (*p* < 0.05), with 95% confidence intervals (CI) calculated for odds ratios (OR) to assess the precision of effect estimates.

## Results

### Demographic characteristics

The study population comprised 426 children, equally divided into 213 MIH cases and 213 non-MIH controls, with a balanced sex distribution (50% male, 50% female) across both groups (Table 1). The mean age of the children was 8.4 ± 1.4 years, with no significant difference between groups (MIH: 8.3 ± 1.5 years; non-MIH: 8.4 ± 1.4 years; t = 0.632, *p* = 0.528). Maternal age at birth ranged from 18 to 49 years, with a mean of 28.8 ± 4.9 years, showing no significant difference (MIH: 29.0 ± 5.0 years; non-MIH: 28.5 ± 4.9 years; t = 0.749, *p* = 0.456). The homogeneity in demographic profiles suggests minimal confounding by age or sex.

Table 1 demonstrates that the MIH and non-MIH groups were comparable in age and sex distribution, with p-values > 0.05 indicating no significant differences. This balance ensures that demographic factors do not skew the analysis of risk factors.

**Table 1:** Demographic characteristics of MIH and Non-MIH groups

Characteristic	MIH (*n* = 213)	Non-MIH (*n* = 213)	Test Statistic	*p*-value
Age (mean ± SD, years)	8.3 ± 1.5	8.4 ± 1.4	t = 0.632	0.528
Sex (male)	107 (50.2%)	107 (50.2%)	χ² = 0.000	1.000
Maternal Age (mean ± SD, years)	29.0 ± 5.0	28.5 ± 4.9	t = 0.749	0.456
Residence (urban)	180 (84.5%)	182 (85.4%)	χ² = 0.076	0.783
Parental Education (≥ high school)	152 (71.4%)	156 (73.2%)	χ² = 0.198	0.656
Birth Order (first-born)	98 (46.0%)	102 (47.9%)	χ² = 0.174	0.676
Family Size (mean ± SD, children)	2.2 ± 0.8	2.1 ± 0.7	t = 1.071	0.285

### Perinatal and postnatal risk factors

For certain risk factors, the sample assessed was reduced to 105 per group for perinatal factors and 108 per group for postnatal factors due to incomplete or unverifiable medical records or maternal recall data, as detailed in the Methods section. Table 2 details the prevalence of perinatal and postnatal risk factors among the 213 MIH cases. Perinatal factors included vaginal delivery (45, 42.9% of 105 assessed), preterm birth (≤ 37 weeks) (19, 18.1% of 105), and hypoxia at birth (7, 6.7% of 105). Postnatal factors included breastfeeding (94, 87.0% of 108 assessed), analgesic use (71, 65.7% of 108), and recurrent diarrhea (40, 37.0% of 108). Other notable conditions were chickenpox (34, 31.5% of 108), hospital admission (31, 28.7% of 108), tonsillitis (12, 11.1% of 108), and vitamin D deficiency (7, 6.5% of 108). Percentages are based on the subset of 105 or 108 children assessed for each factor.

Table 2 provides a comprehensive overview of risk factors in MIH cases, combining perinatal (e.g., preterm birth) and postnatal (e.g., analgesic use) factors. The varying sample sizes reflect the different aspects assessed within the total cohort of 213 MIH cases.

**Table 2:** Prevalence of perinatal and postnatal risk factors in the MIH group (*n* = 213)

Risk Factor	Frequency (*n*)	Percentage (%)	Sample Assessed (*n*)
Vaginal Delivery	45	42.9	105
Preterm Birth (≤ 37 weeks)	19	18.1	105
Hypoxia at Birth	7	6.7	105
Breastfeeding	94	87.0	108
Analgesic Use	71	65.7	108
Recurrent Diarrhea	40	37.0	108
Chickenpox	34	31.5	108
Hospital Admission	31	28.7	108
Tonsillitis	12	11.1	108
Recurrent High Fever (> 39 °C)	10	9.3	108
Otitis Media	8	7.4	108
Vitamin D Deficiency	7	6.5	108
Low Birth Weight (≤ 2500 g)	9	8.6	105

Chi-square analyses (Table 3) identified significant group differences for several factors. Preterm birth was more frequent in the MIH group (18.1% vs. 8.6%, χ² = 6.071, *p* = 0.048, Cramér’s V = 0.171), while vaginal delivery was less common (42.9% vs. 62.9%, χ² = 8.428, *p* = 0.004, Cramér’s V = 0.200). Analgesic use (65.7% vs. 57.4%, χ² = 6.778, *p* = 0.034, Cramér’s V = 0.171) and recurrent diarrhea (37.0% vs. 32.4%, χ² = 6.802, *p* = 0.033, Cramér’s V = 0.171) were significantly higher in MIH, though effect sizes were weak (Phi = 0.177 for both). Chickenpox showed a trend toward significance (31.5% vs. 17.6%, χ² = 5.690, *p* = 0.058, Cramér’s V = 0.163). Vitamin D deficiency (6.5% vs. 8.3%, χ² = 0.375, *p* = 0.829) and breastfeeding < 6 months (4.6% vs. 8.3%, χ² = 2.194, *p* = 0.700) showed no significant differences.

Table 3 highlights significant associations for preterm birth, vaginal delivery, analgesic use, and diarrhea with MIH. Chickenpox approaches significance, suggesting a potential protective role. Weak effect sizes indicate that while statistically significant, these factors may have limited clinical impact individually.

**Table 3:** Comparison of selected perinatal and postnatal risk factors between MIH and Non-MIH groups

Risk Factor	MIH (*n* assessed)	Non-MIH (*n* assessed)	χ²	*p*-value	Cramér’s V
Preterm Birth (≤ 37 weeks)	19 (18.1%, 105)	9 (8.6%, 105)	6.071	0.048	0.171
Vaginal Delivery	45 (42.9%, 105)	66 (62.9%, 105)	8.428	0.004	0.200
Analgesic Use	71 (65.7%, 108)	62 (57.4%, 108)	6.778	0.034	0.171
Recurrent Diarrhea	40 (37.0%, 108)	35 (32.4%, 108)	6.802	0.033	0.171
Chickenpox	34 (31.5%, 108)	19 (17.6%, 108)	5.690	0.058	0.163
Vitamin D Deficiency	7 (6.5%, 108)	9 (8.3%, 108)	0.375	0.829	0.042
Breastfeeding < 6 months	5 (4.6%, 108)	9 (8.3%, 108)	2.194	0.700*	0.068

*Adjusted for breastfeeding duration categories (< 6, 6–12, 12–18, > 24 months)

#### Logistic regression analysis

Multivariate logistic regression results (Table 4) identify key predictors of MIH status. Vaginal delivery was protective, reducing MIH odds by 46% (OR = 0.54, 95% CI: 0.30–0.97, *p* = 0.037). Absence of chickenpox history increased MIH odds (OR = 2.12, 95% CI: 1.40–3.74, *p* = 0.040), indicating a protective effect of chickenpox. Preterm birth (OR = 2.05, 95% CI: 0.86–4.93, *p* = 0.108), analgesic use (OR = 1.63, 95% CI: 0.86–3.09, *p* = 0.131), and recurrent diarrhea (OR = 0.99, 95% CI: 0.50–1.93, *p* = 0.982) were not significant after adjustment. Breastfeeding < 6 months showed no notable effect (OR = 1.45, 95% CI: 0.91–2.31, *p* = 0.117).

Table 4 underscores vaginal delivery and chickenpox history as significant predictors of MIH status. The protective effect of vaginal delivery (46% reduced odds) and the increased risk with no chickenpox history (over twofold odds) suggest distinct roles in MIH etiology. Other factors, despite univariate significance, did not retain importance in the adjusted model, indicating potential confounding or limited independent effects.

**Table 4:** Multivariate logistic regression analysis of risk factors for MIH

Variable	Odds Ratio (OR)	95% CI	*p*-value
Preterm Birth (≤ 37 weeks)	2.05	0.86–4.93	0.108
Vaginal Delivery	0.54	0.30–0.97	0.037
No Chickenpox History	2.12	1.40–3.74	0.040
Analgesic Use	1.63	0.86–3.09	0.131
Recurrent Diarrhea	0.99	0.50–1.93	0.982
Breastfeeding < 6 months	1.45	0.91–2.31	0.117

## Discussion

This case-control study of 426 children aged 6–12 years investigated the perinatal and postnatal risk factors associated with Molar-Incisor Hypomineralization (MIH), comparing those with MIH to healthy controls. The findings revealed vaginal delivery and a history of chickenpox as significant protective factors against MIH, while preterm birth, frequent analgesic use, and recurrent diarrhea showed modest associations that weakened in multivariate analysis. Other factors, such as hypoxia at birth, vitamin D deficiency, and short breastfeeding duration, exhibited no consistent links to MIH. Demographic characteristics, including age, sex, and maternal age, were balanced across groups, minimizing potential bias in the analysis. These results contribute to the growing body of evidence on MIH’s multifactorial etiology, highlighting the interplay of perinatal and postnatal factors with potential regional variations that warrant further exploration [21].

The link between analgesic use and MIH aligns with prior research suggesting that medications in early childhood may interfere with enamel formation. Studies have proposed that analgesics, particularly those commonly used for fever or pain, could disrupt ameloblast activity during critical developmental stages [22]. However, this association weakened when other factors were considered, hinting that illnesses prompting analgesic use might play a role. This suggests a need to explore specific medication types and their timing in future research. For instance, a study found that paracetamol exposure in the first year of life was associated with increased MIH risk, potentially due to its impact on oxidative stress in ameloblasts. Similarly, non-steroidal anti-inflammatory drugs (NSAIDs) have been implicated in enamel defects in animal models, suggesting a dose-dependent effect that could be relevant to human studies [23].

Recurrent diarrhea as a potential MIH risk factor echoes findings from studies in other regions, where gastrointestinal issues have been tied to enamel defects, possibly due to mineral malabsorption [24]. The modest effect observed here implies that while diarrhea may contribute, its impact might be limited or influenced by nutritional status. This could connect to the slight association with shorter breastfeeding, where early weaning might reduce mineral availability during enamel development. A cohort study reported that children with recurrent gastrointestinal issues in early childhood had a higher prevalence of MIH, possibly due to disrupted calcium absorption, underscoring the need to investigate dietary interventions [25].

The protective effect of chickenpox against MIH stands out as unexpected, contrasting with much of the global literature. Previous studies have often linked viral infections, including chickenpox, to increased MIH risk, suggesting systemic stress as a trigger [26]. While the univariate analysis showed a marginally non-significant association, suggesting the possibility of chance, the multivariate regression confirmed a significant protective effect, indicating that the absence of chickenpox history increases MIH odds after adjusting for confounders. This study’s finding raises the possibility that chickenpox might bolster ameloblast resilience through an immune response, a novel idea that challenges existing assumptions and calls for further investigation. For example, a study suggested that early viral exposures may modulate immune responses in ways that protect against certain developmental defects, potentially through cytokine-mediated pathways [27]. This hypothesis aligns with emerging research on the hygiene hypothesis, which posits that early microbial exposures may enhance immune regulation, potentially influencing enamel-forming cells.

The higher prevalence of vitamin D deficiency in the non-MIH group contradicts common hypotheses tying nutritional deficits to MIH. Research elsewhere has suggested that inadequate vitamin D impairs enamel mineralization [28], yet this study’s result might reflect widespread supplementation practices or dietary differences in the region. This discrepancy underscores how nutritional factors in MIH etiology can vary across populations. Antihistamine use and otitis media being more frequent in the non-MIH group diverge from studies associating respiratory issues and related medications with MIH [29]. This could imply that such conditions don’t consistently affect enamel development or that their timing relative to amelogenesis varies. Alternatively, antihistamines might dampen inflammation linked to MIH risk, offering a new angle for exploration.

The absence of a clear link between recurrent high fever and MIH differs from evidence suggesting pyrexia disrupts ameloblast function [30]. This might indicate that fever’s role depends on its severity or timing, with too few cases in this study to show a strong effect. Similarly, other infections like tonsillitis showed no clear connection, suggesting systemic inflammation may not be a dominant factor globally. In addition, the slight association between shorter breastfeeding duration and MIH supports the idea that early nutrition influences enamel quality. Studies worldwide have found that prolonged breastfeeding may lower MIH risk by ensuring mineral supply during tooth formation [31]. This finding hints that early dietary transitions could heighten susceptibility, though its modest impact suggests other factors are at play.

The protective role of vaginal delivery, reducing MIH odds by 46%, is a novel finding that merits further exploration. Cesarean delivery, increasingly common globally, may alter early microbial colonization and immune development, potentially influencing enamel formation [32]. A systematic review found that cesarean-born children have distinct oral microbiomes, which may affect enamel mineralization through altered immune responses or microbial interactions [33]. This could explain the observed protective effect of vaginal delivery, as exposure to maternal vaginal microbiota may enhance immune regulation during critical developmental periods. Additionally, perinatal stress associated with cesarean delivery, such as hypoxia or hormonal fluctuations, may disrupt ameloblast activity, as suggested by animal studies [34]. These findings highlight the need for longitudinal studies to examine delivery mode’s long-term impact on dental health, particularly in regions with high cesarean rates like Iran.

Meanwhile, Environmental factors, such as pollution or toxin exposure, weren’t directly measured but could shape MIH risk, as noted in global research [35]. Industrial regions often report higher MIH prevalence, yet this study focused on medical and nutritional factors. The low occurrence of respiratory conditions like asthma in both groups might reflect under-reporting or unique regional traits, differing from urban centers with higher MIH rates.

The absence of a clear link between recurrent high fever or hypoxia at birth and MIH differs from evidence suggesting pyrexia or oxygen deprivation disrupts enamel formation [36]. This may indicate that severity, duration, or developmental timing of these stressors was insufficient to impact MIH in this cohort. Environmental factors, such as pollution or toxin exposure, were not directly assessed but could shape MIH risk, as noted in industrial regions with higher prevalence [35]. The low occurrence of conditions like asthma or tonsillitis in both groups might reflect regional health patterns, contrasting with urban centers where respiratory issues correlate with MIH.

These findings illustrate MIH’s complex etiology, where perinatal and postnatal factors intertwine with genetic and environmental influences. The protective roles of vaginal delivery and chickenpox, alongside modest links to preterm birth, analgesics, and diarrhea, suggest that systemic and developmental responses are pivotal. Comparisons with worldwide studies reveal both consistencies (e.g., perinatal stressors) and discrepancies (e.g., chickenpox’s role), emphasizing the value of region-specific data. Future research should employ prospective designs with biological markers (e.g., enamel protein analysis, immune profiles) and environmental analyses (e.g., heavy metal exposure) to clarify these patterns. Larger, longitudinal studies could disentangle the timing and cumulative effects of these factors, guiding targeted prevention strategies that span prenatal care, delivery practices, and early childhood health management.

### Limitations

This study has several strengths that enhance its contribution to MIH research. The large sample size (*n* = 426) provided sufficient power to detect associations, and the inclusion of participants from both a university dental clinic and public schools ensured a diverse, representative sample from Arak City. The use of a validated 48-item checklist (Cronbach’s α = 0.85) for data collection, combined with face-to-face interviews by trained interviewers, improved the reliability of maternal recall. Additionally, MIH diagnoses were conducted by three calibrated pediatric dentists using standardized EAPD criteria, achieving high inter-examiner reliability.

This study has notable Limitations. Its case-control design cannot establish causality, relying on parental recall, which may introduce bias or inaccuracies in reporting postnatal events. The reliance on maternal recall for early life exposures, covering events up to 12 years in the past depending on the child’s age, poses a significant risk of recall bias, particularly for details like medication use or illness history. While cross-verification with medical records and school health logs was performed where available, not all responses could be verified, and differential recall between MIH and non-MIH groups may have influenced results. Sensitivity analyses excluding unverifiable responses were not conducted due to the small proportion of unverifiable data and logistical constraints, but such analyses could enhance robustness in future studies. The use of trained but non-calibrated dental hygienists for initial screening at schools may have led to overlooking mild MIH cases, as subtle opacities require expert assessment. However, referral of all suspected cases for examination by calibrated dentists likely minimized this risk. The assessment of vitamin D deficiency based on historical diagnoses or maternal reports, without contemporaneous blood samples, may further limit accuracy, as underdiagnosis or misreporting could affect prevalence estimates.The sample, though sufficient, came from a single region, limiting its applicability to broader populations in Iran or globally with varying environmental and healthcare contexts. Socioeconomic status was broadly assessed via parental education, potentially missing finer economic influences on nutrition or health access. Environmental factors, like pollution, weren’t evaluated despite their possible role in MIH. Lastly, focusing on postnatal factors may have overlooked prenatal or perinatal contributors that could affect the observed associations. Additionally, incomplete or unverifiable data for certain perinatal and postnatal risk factors resulted in reduced sample sizes (105 or 108 per group) for specific analyses, which may limit the generalizability of findings for those variables.

## Conclusion

The results underscore the need for a nuanced approach to MIH etiology, as the weak effect sizes of many factors indicate that no single exposure dominates its development. Instead, cumulative or synergistic effects across the perinatal and early childhood periods may be at play, necessitating broader investigation. Recommendations for future research include longitudinal cohort studies to track perinatal and postnatal exposures over time, integrating genetic profiling to explore susceptibility markers and environmental factors such as pollution, heavy metal exposure, or dietary deficiencies. Larger, multicenter studies could further clarify the roles of weakly associated factors like analgesic use and diarrhea, potentially identifying dose-response relationships or critical exposure windows.

Clinically, these findings suggest practical implications for MIH prevention. Monitoring preterm infants and those born via cesarean delivery for early dental screening could facilitate timely intervention, while cautious use of analgesics in early childhood and proactive management of gastrointestinal health (e.g., recurrent diarrhea) may mitigate risk. Encouraging vaginal delivery where medically feasible and optimizing neonatal care to reduce perinatal stress could also play a preventive role. Public health strategies should prioritize maternal and child health education, emphasizing breastfeeding support, infection management (e.g., understanding the balance of childhood illnesses like chickenpox in the context of vaccination), and nutritional adequacy to address modifiable factors. Tailoring these approaches to regional data, such as the specific obstetric and health patterns in Arak City, Iran, will enhance their effectiveness. Collectively, this study lays a foundation for refining MIH prevention strategies, urging a holistic perspective that bridges prenatal care, neonatal outcomes, and early childhood health.

## Data Availability

The datasets used and/or analyzed during the current study are available from the corresponding author on reasonable request.

## Abbreviations

MIH: Molar-incisor hypomineralization
EAPD: European academy of paediatric dentistry
SD: Standard deviation
OR: Odds ratio
